# Complex energies of the coherent longitudinal optical phonon–plasmon coupled mode according to dynamic mode decomposition analysis

**DOI:** 10.1038/s41598-021-02413-w

**Published:** 2021-11-30

**Authors:** Itsushi Sakata, Takuya Sakata, Kohji Mizoguchi, Satoshi Tanaka, Goro Oohata, Ichiro Akai, Yasuhiko Igarashi, Yoshihiro Nagano, Masato Okada

**Affiliations:** 1 Department of Physics, University of Tokyo, 7-3-1 Hongo, Bunkyo-ku, Tokyo, 113-0033 Japan; 2 Department of Physical Science, Osaka Prefecture University, Gakuen-cho 1-1, Sakai, 599-8531 Japan; 3 Institute of Pulsed Power Science, Kumamoto University, 2-39-1 Kurokami Chuo-ku, Kumamoto, 860-8555 Japan; 4 Graduate School of System and Information Engineering, University of Tsukuba, Tsukuba, Ibaraki 305-8577 Japan; 5 Technology Agency, PRESTO, Kawaguchi, Saitama 332-0012 Japan; 6 Department of Complexity Science and Engineering, Graduate School of Frontier Sciences, University of Tokyo, 5-1-5 Kashiwanoha, Kashiwa, Chiba 277-8561 Japan

**Keywords:** Ultrafast photonics, Characterization and analytical techniques, Quantum optics, Nonlinear optics

## Abstract

In a dissipative quantum system, we report the dynamic mode decomposition (DMD) analysis of damped oscillation signals. We used a reflection-type pump-probe method to observe time-domain signals, including the coupled modes of long-lived longitudinal optical phonons and quickly damped plasmons (LOPC) at various pump powers. The Fourier transformed spectra of the observed damped oscillation signals show broad and asymmetric modes, making it difficult to evaluate their frequencies and damping rates. We then used DMD to analyze the damped oscillation signals by precisely determining their frequencies and damping rates. We successfully identified the LOPC modes. The obtained frequencies and damping rates were shown to depend on the pump power, which implies photoexcited carrier density. We compared the pump-power dependence of the frequencies and damping rates of the LOPC modes with the carrier density dependence of the complex eigen-energies of the coupled modes by using the non-Hermitian phenomenological effective Hamiltonian. Good agreement was obtained between the observed and calculated dependences, demonstrating that DMD is an effective alternative to Fourier analysis which often fails to estimate effective damping rates.

## Introduction

In recent years, non-Hermitian Hamiltonians have been used to study dissipative effects in various quantum phenomena^1–7^, which these phenomena are characterized by exponential damping because they break time symmetry^8^. The time symmetry breaking seems to contradict the principle of microscopic mechanics, which states that the time evolution of physical systems is time-reversible assuming that it is unitary. Therefore, a fundamental challenge has been to find a consistent interpretation of irreversible phenomena within a unified theoretical framework^9–11^. A Hamiltonian in a Hilbert space cannot be the time evolution generator of a system representing dissipation. Instead, non-Hermitian effective Hamiltonians have been derived to describe open quantum systems with dissipation, including the environment^12–16^.

Time symmetry has been observed to break in mesoscopic quantum systems and optical systems^17–24^. In particular, the Fano resonance has attracted for breaking time symmetry. The Fano resonance is typically interpreted as quantum interference^25^. On the other hand, because it inevitably involves dissipation, the Fano resonance has been reinterpreted in terms of a non-Hermitian Hamiltonian^26,27^. Motivated by these observations, we focused on the coherent longitudinal optical phonon–plasmon coupled mode, an open quantum system with dissipation similar to Fano resonance^28,29^. This is called the longitudinal optical phonon-plasmon coupled (LOPC) mode, which can be experimentally observed as a coherent phonon with the pump-probe method. In the LOPC mode, the observed signal shows damped oscillations resulting from dissipation.

In the experiment, we observe a 1D time-series signal, which is usually subjected to Fourier analysis. The Fourier transformed spectra of observed damped oscillation signals also show broadened and asymmetric modes, making it difficult to evaluate their frequencies and damping rates. However, plane wave expansion cannot represent exponential damping, so the Fourier transform is not suitable for exponential damping analysis because it is a plane wave expansion. In contrast, dynamic mode decomposition (DMD) is directly applicable to exponential damping analysis.

In this study, we propose a method of analysis using dynamic mode decomposition (DMD) to estimate the damping rate for exponential damping. DMD has previously been applied to extracting modes with decay and growth from time-series data^30–32^. Schmid et al.^33^ proposed applying DMD to hydrodynamics. DMD has a wide range of applicability in fields such as neuroscience and nonlinear systems^34,35^. In recent years, DMD has been applied to coherent phonon spectroscopy^36,37^. Thus, DMD is widely used for the analysis of complex time series that include damping. Because it represents data as a sum of vibration components with damping oscillations, it can be regarded as a natural extension of the Fourier transform. Thus, DMD is a suitable method for damped vibration analysis.

We present a framework for estimating the damping rate of the LOPC mode, for which previous studies only provided a heuristic method^38,39^. Thanks to the ability of DMD to estimate the dumping rate of the LOPC modes, we found that coherent phonon measurements of LOPC are consistent with the results calculated from the complex eigenvalues of the phenomenological non-Hermitian effective Hamiltonian. The results suggest that the LOPC coupling state can be interpreted by a non-Hermitian effective Hamiltonian.

## Results

Here, we present the results of coherent phonon signal analysis of the LOPC mode using DMD. The LOPC mode can be divided into an upper branch (UB) and lower branch (LB) for non-Hermitian effective Hamiltonians. The two modes show damped oscillations originating from dissipation, so the peaks of the Fourier spectrum are broadened. The peaks in the UB are difficult to find because of their weak intensity. By using DMD, we can precisely estimate the frequency and damping rate, and we can compare the estimated mode profiles with the phenomenological non-Hermitian effective Hamiltonian. By comparing the frequency and damping rate estimated by DMD against the carrier density, we obtain an antisymmetric relationship for each eigenstate. This relationship is the same as the variation in the energy eigenvalue of the phenomenological non-Hermitian effective Hamiltonian for the carrier density and follows the results estimated by DMD.

### Effective Hamiltonian and coherent phonon

**Figure 1:**
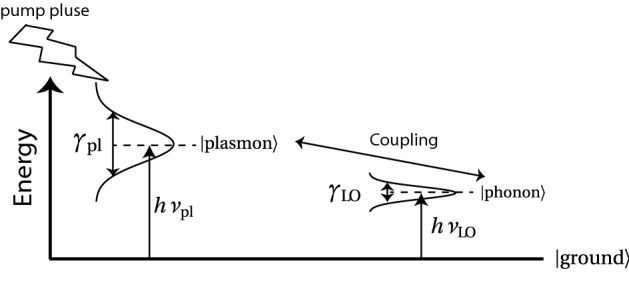
Energy diagrams of interactions between discrete levels with relaxation.

In this section, we describe the Hamiltonian and exponential damping forms of the LOPC mode. Figure 1 shows the semi-classical representation of the coupling between fast-relaxing plasmons and slow-relaxing LO photon as two coupled transient states in which the energy widths are related to the damping rates. Yokota^40^ previously proposed a transient oscillation model of the interaction between a photo-excited non-equilibrium plasma and GaAs lattice for representing the LOPC mode at the femtosecond timescale. This model uses the classical simultaneous equations of motion without relaxation to represent the frequencies of the LOPC modes as follows:

1 $$\begin{aligned} \nu ^2_{\pm } = \frac{1}{2} \left( \nu ^2_{\text {pl}} + \nu ^2_{\text {LO}} \pm \sqrt{ \left( \nu ^2_{\text {pl}} + \nu ^2_{\text {LO}}\right) - 4 \nu ^2_{\text {pl}} \nu ^2_{\text {TO}} }\right) , \end{aligned}$$

where $\nu _{\text {pl}}, \nu _{\text {LO}},$ and $\nu _{ \text {TO}}$ are the plasma frequency, LO phonon frequency and transverse optical (TO) phonon frequency, respectively. $\nu _{+}$ and $\nu _{-}$ are the UB and LB frequencies, respectively, of the LOPC mode. Many papers have discussed the relationship between the dispersion relation according to the classical equations of motion with relaxation and the results obtained by Raman spectroscopy and the pump-probe method^41–43^. In recent years, there has been a growing momentum to understand the dispersion relation from a microscopic perspective by using Hamiltonians.

In this study, we analyze the LOPC modes using a non-Hermitian effective Hamiltonian that can express the effects of damping, rather than classical models. Figure 1 shows a phenomenological non-Hermitian effective Hamiltonian for expressing the coupling between LO phonons with a small damping rate and plasmons with a large damping rate. Hereafter, we refer to the phenomenological non-Hermitian effective Hamiltonian as a “phenomenological effective Hamiltonian”. Singwi and Tosi^44^ formulated the Hamiltonian for coupled plasmons and phonons:

2 $$\begin{aligned} H= & {} h \nu _{\text {pl}} a^{\dagger } a + h \nu _{\text {LO}} b^{\dagger } b + h C \left( a^{\dagger } b + a b^{\dagger }\right) , \end{aligned}$$

3 $$\begin{aligned} C= & {} \frac{1}{2} \left( {\hat{\nu }}_{\text {pl}} {\hat{\nu }}_{\text {LO}} \left( 1 - \frac{{\hat{\nu }}^2_{\text {LO}}}{{\hat{\nu }}^2_{\text {TO}}}\right) \right) ^{1/2}, \end{aligned}$$

where $\nu _{\text {pl}}$ and $\nu _{\text {LO}}$ are the frequencies of the plasmons and LO phonons, respectively; $a^{\dagger }$ and *a* are the creation-annihilation operators related to the plasmons; $b^{\dagger }$ and *b* are the creation-annihilation operators related to the LO phonons; *h* is planck constant; and *C* is the coupling constant. To consider relaxation and dissipation in the phenomenological effective Hamiltonian. We can introduce an imaginary part for the frequencies of the phonons and plasmons:

4 $$\begin{aligned} {\hat{\nu }}_{\text {pl}}&= \nu _{ \text {pl}} - i \gamma _{\text {pl}}, \end{aligned}$$

5 $$\begin{aligned} {\hat{\nu }}_{ \text {LO}}&= \nu _{ \text {LO}} - i \gamma _{ \text {LO}}, \end{aligned}$$

6 $$\begin{aligned} {\hat{\nu }}_{ \text {TO}}&= \nu _{ \text {TO}} - i \gamma _{ \text {TO}}. \end{aligned}$$

where, ${\hat{\nu }}_{ \text {pl}},{\hat{\nu }}_{ \text {LO}}$ and ${\hat{\nu }}_{ \text {TO}}$ are the complex frequencies of the plasmon, LO phonon and TO phonon, respectively. Each complex frequency is expressed in terms of the vibration frequency component of the real part ($\nu _{ \text {pl}},\nu _{ \text {LO}}$ and $\nu _{ \text {TO}}$) and the damping component of the imaginary part ($\gamma _{ \text {pl}},\gamma _{ \text {LO}}$ and $\gamma _{ \text {TO}}$). Many researchers have reported that the relaxation processes of the plasmon are due to electron-electron scattering, electron-phonon scattering, Landau damping and so on^45–47^. We assumed that the imaginary part of the complex frequency of the plasmon (i.e., the damping rate of the plasmon) depends on carrier density $n_{\text {pl}}$^46^:

7 $$\begin{aligned} \gamma _{ \text {pl}} = \gamma _{ \text {pl0}} + c n_{\text {pl}}^{1/3}, \end{aligned}$$

where $\gamma _{ \text {pl0}}$ is the density-independent term and *c* is the proportionality coefficient. This equation means that the damping rate of plasmon is proportional to the one-third power of the carrier density. The effective Hamiltonian *H* with the complex frequencies of Eqs. (4)–(6) obtains two LOPC eigenmodes with complex eigenfrequencies, $z_n=\nu _n - i\gamma _n$, where the real and imaginary parts denote the renormalized frequency shifts and decay rates of the eigenmodes, respectively^48^. The LB and UB are identified by the lower and upper real values, respectively, of the complex eigenfrequencies.

Next, we show that the LOPC mode described by the phenomenological effective Hamiltonian can be observed as a signal of damped oscillations. The pump-probe method can be used to observe damping modes due to LOPC modes reflecting the material’s polarization. When the central wavelength of the pulse laser is tuned to the band edge of n-GaAs, the Franz–Keldysh effect leads to modulation of the optical interband transition with a nonlinear dependence on the macroscopic electric field associated with coherent phonons^49^. In the pump-probe method, the probe pulse can be used to observe the time variation of the polarization caused by the LOPC mode induced by the pump pulse. According to previous studies^50–52^, the observed change in reflectivity can be written as the following $\chi _3$ process;

8 $$\begin{aligned} \left( \frac{\Delta R}{R_0}\right)&\propto - \frac{ {\text {Im}}\left[ \Delta P \cdot E_{\text {pr}}^{*} \right] }{\left| E_{\text {pr}}\right| ^{2}}, \end{aligned}$$

9 $$\begin{aligned} \Delta P&= \chi _3 E_{\text {pu}} E^*_{\text {pu}} E_{\text {pr}}. \end{aligned}$$

*P* is the polarization caused by the pump light: $\Delta P \propto \sum _n P_{0n} \exp (- i z_n t)$, where, $\chi _3$ is the third-order nonlinear susceptibility and $z_n$ is the eigenvalue from the phenomenological effective Hamiltonian. $E_{\text {pr}}$ is the electric field of the probe light and $E_{\text {pu}}$ is the electric field of the pump light. This represents the observation of changes due to the interaction between phonons and polarization in the material. The observed signal is the sum of the damped oscillations because $z_n$ is complex

10 $$\begin{aligned} \Delta R (t) /R_0 \propto \sum _{n} \exp \left( - {\gamma _{n}} t\right) \left( {\varvec{a}}_{n} \cos \nu _{n} t + {\varvec{b}}_{n} \sin \nu _{n} t \right) \end{aligned}$$

where ${\varvec{a}}$ and ${\varvec{b}}$ are amplitudes.

### Measurements and fourier analysis

As an experiment to measure the LOPC mode in n-type GaAs semiconductors, reflection-type pump-probe measurements were performed at room temperature. We used a pulsed laser with central energy of about 1.59 eV and a pulse width of about 80 fs. The amount of doping in the semiconductor sample ($n_{\text {dope}}$) was $3 \times 10^{17} {\text {cm}}^{-3}$. The carrier density $n_{ \text {pl}}$ related to the plasma frequency ($\nu _{ \text {pl}}$) in the sample depended on the amount of doping ($n_{\text {dope}}$) and the excited carrier density ($n_{\text {exc}}$)^53^ as Drude model^54^:

11 $$\begin{aligned} n_{ \text {pl}}&= n_{\text {dope}} + n_{\text {exc}}, \end{aligned}$$

12 $$\begin{aligned} \nu _{ \text {pl} }&= \left( \frac{e^2 n_{\text {pl}}}{m_e \epsilon _0 \epsilon _r}\right) ^{1/2} \end{aligned}$$

where *e* is elementary charge; $m_e$ is mass of the electron; $\epsilon _0$ is vacuum permittivity; and $\epsilon _r$ is relative permittivity. We measured the dependence on the carrier density $\nu _{ \text {pl} }$ of the LOPC mode at different pump-power densities. The samples were at various pump-power densities and extracted only the vibrational components were extracted from the changes in reflectivity over time that were obtained by the reflection-type pump-probe measurements.

**Figure 2:**
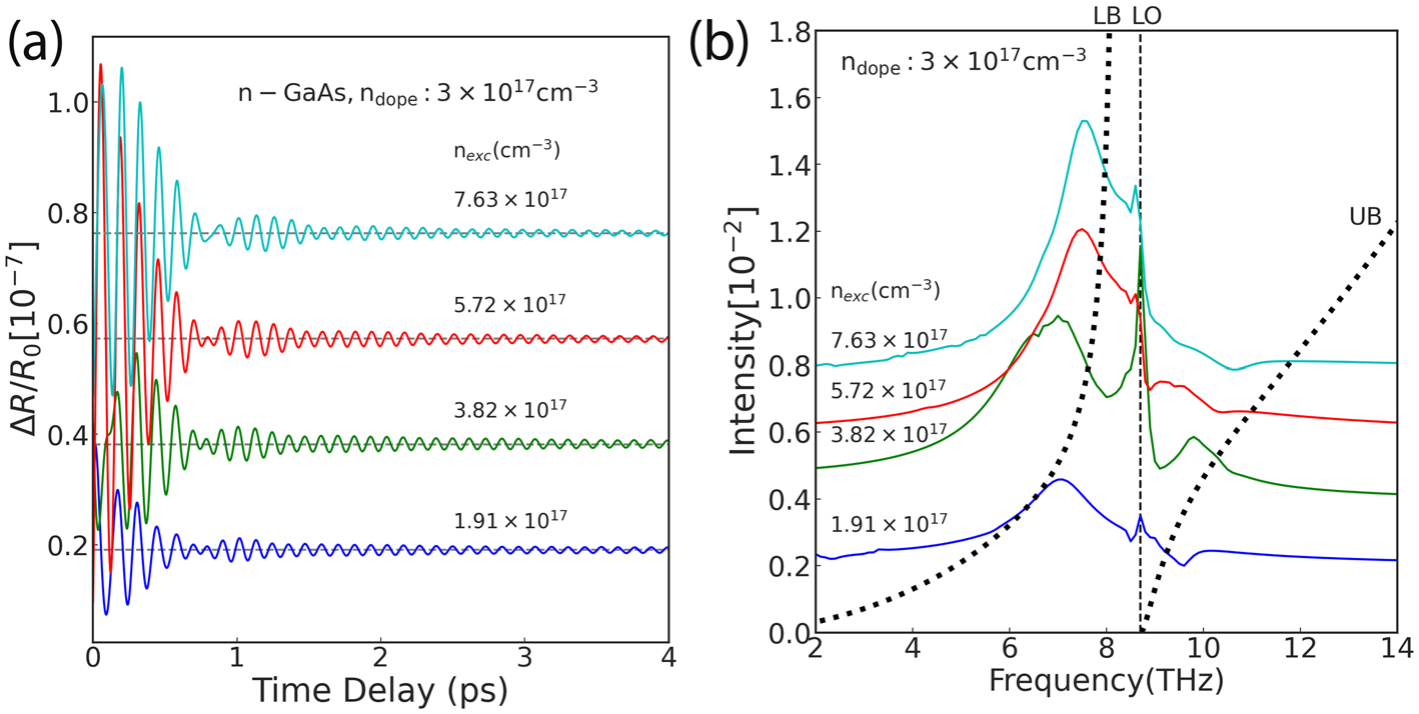
(**a**) Oscillatory components observed in the time reflectance variation of *n*-type GaAs semiconductors at each carrier density ($n_{exe}$). The horizontal axis shows the time of measurement, and the vertical axis shows the signal strength. The signals are plotted vertically as a function of the carrier density. (**b**) Fourier spectrum of the time-resolved reflectivity change at each carrier density. The horizontal axis indicates the frequency, and the vertical axis indicates the intensity. The Fourier spectra are plotted vertically as a function of the carrier density as in (**a**). The dashed lines of the LB and UB curves in show the upper branch on the high-frequency side and lower branch on the low-frequency side, respectively, of the LOPC mode as calculated from the phenomenological effective Hamiltonian. The thin vertical dashed lines represent the frequencies of LO phonons at 8.7 THz.

Here, we point out the problems with applying Fourier analysis to coherent phonon measurement data. The comparison of the measurement data with the phenomenological effective Hamiltonian required detecting the UB and LB of the LOPC mode and estimating the frequency and damping rate from the measurement signal. Coherent phonon measurement data are obtained as damped oscillations. Figure 2a shows the observed pump-probe signal of GaAs semiconductors for different carrier densities. Each representative signal is color-coded. Figure 2b shows the Fourier spectrum of the observed signals. Applying Fourier analysis to the LOPC mode has two problems; it is difficult to estimate the damping rate from Fourier spectrum^55^, and mode extraction is difficult especially for the UB. Figure 2a shows that the measurement signal is a superposition of damped oscillations, and the peak of the Fourier spectrum is broadened. The spectrum in Fig. 2b shows that the peak structure itself may be undetectable for a UB with small mode amplitudes because of the broadening and asymmetry of the peaks caused by exponential damping. Thus, Fourier analysis is not suitable for analyzing measurement data of non-Hermitian phenomena with dissipation.

### Results of DMD and comparison with model

**Figure 3:**
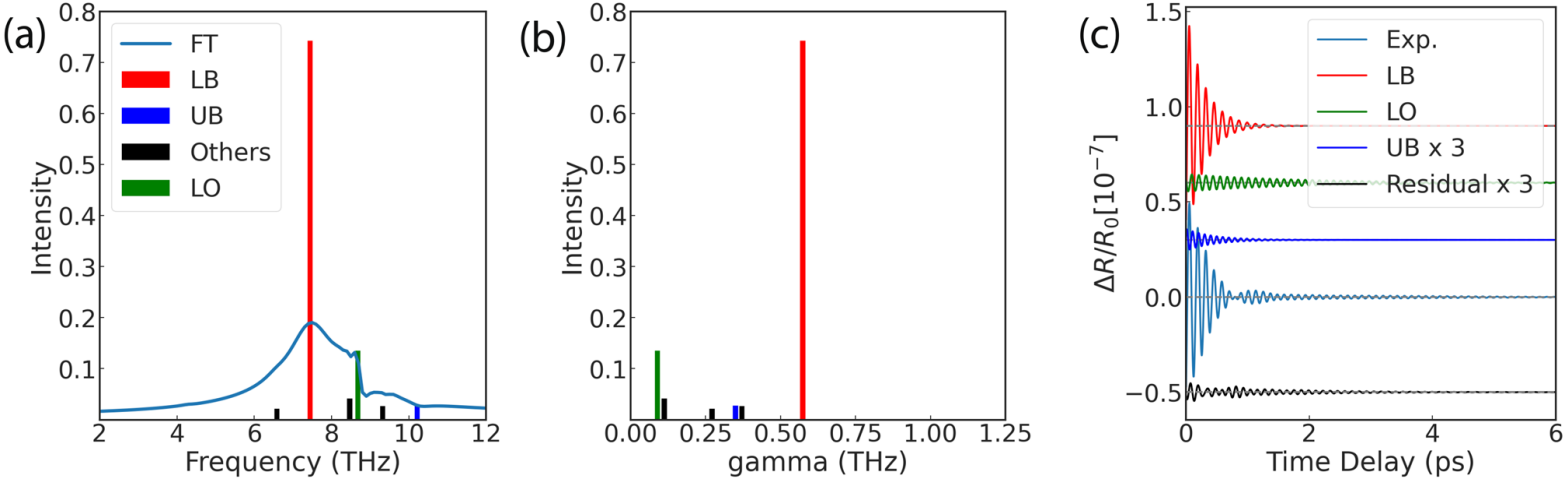
The spectra for the case of $n_{exe} = 5.72 \times 10^{17} {\text {cm}}^{-3}$. (**a**) The results of frequencies. The horizontal axis shows the frequencies, and the vertical axis shows the intensity of each mode of DMD and Fourier spectrum. The black bars indicate the discrete modes obtained by DMD. The green, red, and blue bars indicate the modes corresponding to the LO, LB, and UB modes, respectively, of the black DMD mode. The solid light blue line shows the Fourier spectrum. (**b**) The results of dumping rate ${\tilde{\gamma }}$. The horizontal axis shows $\gamma$, and the vertical axis shows the strength of the DMD mode. Modes selected in (**a**) are colored in the same way. (**c**) Result of restoring the original signal format for each modes. As in (**a**) and (**b**), each mode is color-coded. The solid black lines show the residuals of the sum of the three selected modes and the experimental signal. To visualize the vibration components, we tripled the amplitudes of the UB and residuals.

We applied DMD to analyzing damping oscillation signals as an alternative to Fourier analysis. As an example, Fig. 3 shows the results for $n_{exe} = 5.72 \times 10^{17}\;{\text {cm}}^{-3}$. First, we confirmed that DMD could extract UB modes. Next, we applied DMD to estimating the dumping rate. We then compared the frequency estimation results with those from Fourier analysis. Figure 3a compares the amplitudes of the DMD modes for each mode for the frequency with the Fourier spectrum. The DMD frequencies are plotted as a bar graph because DMD decomposed the signal into discrete modes. The UB, LB, and LO modes are color-coded as blue, red, and green, respectively. The other modes are color-coded as black. To clarify the results, only representative modes with large amplitudes are shown. The LB mode had a strong DMD peak at 8.68 THz. The LO mode also had a DMD peak at 8.68 THz, which was consistent with the Fourier spectrum. Two modes with larger amplitudes were identified at higher frequencies than the LO mode. The high-frequency mode of 10.22 THz was designated as the UB because it was considered a branch of the LOPC and higher than the reference frecuency of 8.7 THz for the LO mode. The DMD successfully extracted the UB peak, which was undetected by the Fourier analysis. Thus, these results suggest that DMD can extract modes that may be missing in Fourier spectra.

An important parameter for the phenomenological effective Hamiltonian is the damping rate, which DMD is able to estimate. Figure 3b shows the damping rate results: it is illustrated in the same manner as Fig. 3a. The damping rates of the UB, LB, and LO modes selected from the intensity of frequency plots were 0.35, 0.57, and 0.09 THz, respectively. The dumping rate is related to the lifetime of each mode; the larger the value, the shorter the lifetime. The UB and LB in the LOPC mode have short lifetimes while the LO mode has a long lifetime. This result is consistent with the finding that the bonding state (i.e., LOPC mode) has a shorter lifetime than the coherent phonon (LO mode). Unlike the Fourier analysis, DMD was able to estimate the damping rate.

The vibration components extracted by DMD can be visualized to help clarify their qualitative nature. Figure 3c shows the results of restoring each mode from Fig. 3a,b to the original signal. The figure shows the UB, LB, and LO modes and the difference between the sum of those three modes and the measurement. We tripled the vibration components of the UB mode and ‘Residual’ for easier visualization. The LB mode indicated a large-amplitude vibration component up to 2 ps, while the LO mode indicated a vibration component that continues after 2 ps. The ‘Residual’ amplitude was small, especially after 2 ps and approached close to zero. The ‘Residual’ vibration component remained for up to 2 ps. This difference was attributed to the effects of fluctuation and experimental artifacts. The small residual indicates that the oscillatory component elements can be explained by three modes: UB, LB, and LO. Thus, DMD is useful for examining the qualitative nature of the modes. Because DMD can estimate the frequency and damping rate, it allows for comparison with the phenomenological effective Hamiltonian.

DMD allows the detection of the UB and LB of the LOPC mode and the estimation of the frequency and damping rate. The real and imaginary parts of the complex eigenvalues calculated from the phenomenological effective Hamiltonian correspond to the frequency and damping rate of the LOPC modes, respectively. So, it allows the complex eigenvalues calculated from the model in Eqs. (5) and (6) be compared with the LOPC modes obtained by DMD. Here, we discuss the dependence on the carrier density of the frequencies and damping rates of the LOPC modes by comparing the calculated eigenvalues with the experimental results.

**Figure 4:**
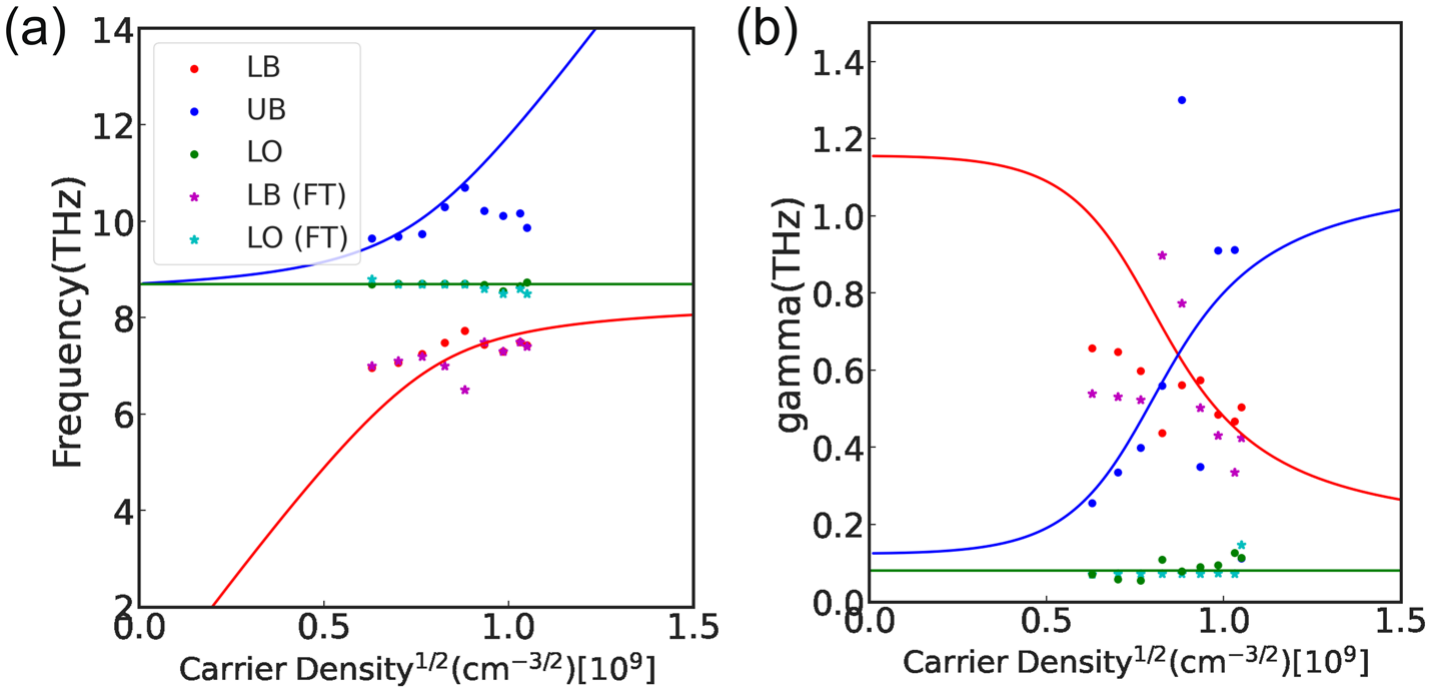
Carrier density dependence of the frequencies and damping rates. The measurement data is one point for each carrier density. For both (**a**) and (**b**), the horizontal axis is $\sqrt{n_{\text {pl}}}$. (**a**) Carrier density dependence of the peak frequencies extracted by DMD and Fourier spectrum (FT). The horizontal axis shows the square root of the carrier density, and the vertical axis shows the frequencies of each mode. Green, blue, and red plots show the LO, UB and LB, respectively. The solid line corresponding to each color shows the carrier density dependence of the real part of the complex energy eigenvalues obtained from Eq. (2,3). Note that the UB of Fourier spectrum is not shown because it was difficult to detect the peak of UB mode from the Fourier spectrum. (**b**) Dependence of the damping rate ${\tilde{\gamma }}$. The Fourier spectrum’s damping rate was estimated from the FWHM of the spectral peaks using the half-power method. The horizontal axis shows the square root of the carrier density, and the vertical axis shows the damping rate ${\tilde{\gamma }}$ of each mode. The plot is color-coded in the same way as (**a**). The solid line shows the carrier density dependence of the imaginary part of Eq. (2,3).

We first discuss the frequency results. Figure 4a illustrates the carrier density dependence of the frequencies obtained by DMD and by the real part of the complex eigenvalue as calculated from the phenomenological effective Hamiltonian. The dots represents the results of experimental data from DMD, and the solid line represents the real part of the calculated complex eigenvalue. The LB, UB, and LO modes are color-coded in the same manner as Fig. 3a. Except for the high carrier density region of the UB mode, the experimental results were in good agreement with the theoretical values of the phenomenological effective Hamiltonian. The deviation in the high carrier density region is because the width of the laser pulse was about $80\; {\text {fs}}$, which made accurate measurement in the high-frequency region difficult. The star points in Fig. 4a show the same frequency result of the Fourier spectrum for comparison. Since it was difficult to detect the peak of UB mode from the Fourier spectrum, UB is not shown in the figure. Although the frequencies for UB and LO of the Fourier spectrum are almost the same as those for DMD, the DMD’s frequencies are closer to the theoretical values.

Next, we discuss the damping rate results. Figure 4b shows the damping rate obtained by DMD and the imaginary part of the phenomenological effective Hamiltonian. The theoretical lines from the non-Hermitian Hamiltonian follow the experimental values extracted from the DMD. The theoretical line of the damping rate $\gamma _{\text {pl}}$ calculated from the phenomenological effective Hamiltonian was derived from Eq. (2,3). Because $\gamma _{\text {pl0}}$ and *c* in Eq. (7) are unknown parameters, in this case, they were determined from the estimated mode properties according to DMD. The previous studies only provided a heuristic approach to estimating these parameters^38,39^. We set $\gamma _{\text {pl0}} = 1.23\;{\text {THz}}$ and $c = 9.61 \times 10^{-5} \; {\text {THz}} \;{\text {cm}}$. We then set the value of $\gamma _{\text {pl0}}$ to minimize the squared error between the estimated value by DMD and theoretical value by the Hamiltonian. We obtained the value of *c* from Hugel et al.^38^ because the value estimated by the least-squares method was very large and deviated from the physically acceptable range. $\gamma _{\text {pl0}}$ was about 1/10 the value by Hugel et al.^38^. Although the estimated parameters did not agree with the results of previous studies, the fitting of the damping rate $\gamma$ in Fig. 4 was generally consistent with the data. Higher accuracy for parameter estimation is a future challenge. We demonstrated that the carrier density dependence of the frequencies and damping rates obtained by DMD can be simultaneously expressed by the complex eigenvalues calculated from the phenomenological effective Hamiltonian. The intercept was $1.23 \; {\text {THz}}$, and the proportionality coefficient was $c = 9.61 \times 10^{-5} \; {\text {THz}} \; {\text {cm}}$. As same for the Fig. 4a, Fig. 4b shows the damping rate of the Fourier spectrum for comparison. The damping rate of the Fourier spectrum was estimated from the full width at half maximum (FWHM) of the Fourier spectral peaks using the half-power method^56,57^. As in Fig. 4a, UB is not shown because it was difficult to find the peak. From the LB modes results, it is clear that the damping rates of DMD are closer to the theoretical values. For LO modes with low dumping rates, the results are almost identical to those of DMD.

## Discussion

In this study, we applied DMD to analyzing exponential damping in quantum dissipative systems. We used the pump-probe method to observe time-domain signals containing coupled LO phonon and plasmon modes at various pump powers. Fourier transformed spectra showed broad and asymmetric modes, which makes it difficult to evaluate their frequencies and damping rates precisely. In contrast, the DMD can directly handle exponential damping and thus can accurately evaluate the frequency and damping rate of damped oscillations. We successfully applied DMD to identifying LOPC modes and clarifying the excitation carrier dependence of the frequency and damping rate. We compared the pump-power dependence of the LOPC modes with the carrier density dependence of the complex eigen energies of the phenomenological effective Hamiltonian. Good agreement was obtained between the observed and calculated dependences.

Previously, the Fourier transform is generally used to analyze time domain signals such as coherent phonons^58–62^. Because the Fourier transform is a plane wave expansion, the damping rate $\gamma$ of the eigenmodes cannot be estimated directly. Previous studies have used the spectral width and shape symmetry^59,60^. However, it is difficult to estimate the damping rate $\gamma$ from the spectral shape is difficult because of experimental artifacts such as background roar effects and measurement noise. In this study, we used DMD instead of the Fourier transform to solve this problem. The form of the modes extracted by the DMD was consistent with the form of the observed signals.

The DMD analysis is an effective alternative to Fourier analysis for estimation of exponential dumping of dissipation phenomenon. The other contribution of our study is to provide a framework for estimating the dumping rate of plasmon mode. In contrast to our proposed framework, the previous studies only provide a heuristic way to estimate the plasmon’s dumping rate^38,39^. It is known that the dumping rate of the plasmon mode is proportional to 1/3 power of the carrier density, and we estimated the intercept and the proportionality coefficient. Although the estimated parameters did not agree with the results of previous studies, the fitting of the damping rate $\gamma$ is generally consistent with the data. Higher accuracy in parameter estimation is a future challenge.

## Method

**Figure 5:**
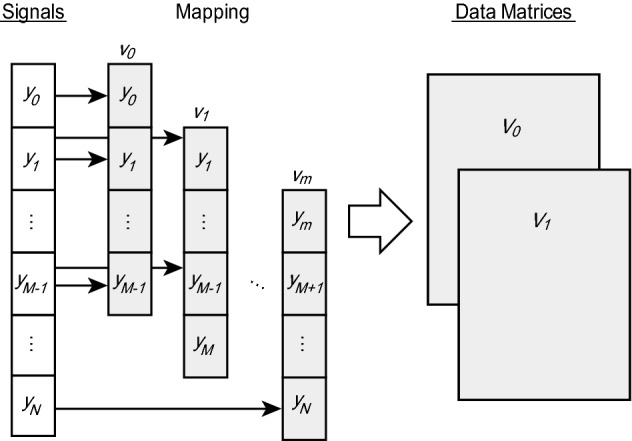
A method of creating a matrix for DMD from the CP signals.

This section explains the DMD algorithm and the form of the damped oscillations expressed by the DMD basis. DMD is a method of decomposing high-dimensional time-series data. However, the measurement signal used in this study is one-dimensional. To apply the DMD to a one-dimensional signal, we constructed a data matrix as shown in Fig. 5, similarly to the previous studies on coherent phonons^36,37^. Now, we can suppose the measurement signal is *N* points with a constant time interval of $\delta t$, and we can denote the time series as $\left( y_0, \ldots , y_N\right)$. We first make a snapshot of the projected measurement signal $y_t$ on a *M* dimensional vector.

13 $$\begin{aligned} {{\varvec{v}}}_t = \left( y_t,y_{t+1},\ldots ,y_{t+M-1}\right) ^{\text {T}}. \end{aligned}$$

We can assume that we obtain $m + 1$ snapshots at regular time intervals $m+1$. Furthermore, the created *M*-dimensional vectors are arranged *m* times to create a data matrix of $M \times m$, where $m = N - M + 1$. We considered the time-shifted pairs ${{\varvec{V}}}_0$ and ${{\varvec{V}}}_1$ as an introduction to the time evolution.

14 $$\begin{aligned} {\varvec{V}}_0&= \left( {\varvec{v}}_0, \ldots , {\varvec{v}}_{m-1}\right) \in {\mathbb {R}}^{M \times m}, \end{aligned}$$

15 $$\begin{aligned} {\varvec{V}}_1&= \left( {\varvec{v}}_1, \ldots , {\varvec{v}}_{m}\right) \in {\mathbb {R}}^{M \times m}. \end{aligned}$$

The objective of DMD is to find a matrix A such that the following relationship holds:

16 $$\begin{aligned} {\varvec{V}}_1 = {\varvec{A}} {\varvec{V}}_0. \end{aligned}$$

here, we briefly describe the DMD algorithm by Jovanović et al.^63^. *A* in Eq. (16) is derived from the least-squares optimization of the following equation

17 $$\begin{aligned} {\varvec{A}} = {\mathop {{{\,\text{arg\,min}\,}}}\limits _{A}} ||{\varvec{V}}_1 - {\varvec{A}} {\varvec{V}}_0||_F = {\varvec{V}}_1 {\varvec{V}}_0^{+} \approx {\varvec{V}}_1 {\varvec{V}} \varvec{\Sigma }^{-1} {\varvec{ U}}^*. \end{aligned}$$

where ${\varvec{P}} \in {\mathbb {R}}^{M \times r}, \varvec{\Sigma } \in {\mathbb {R}}^{r \times r}$, and ${\varvec{Q}} \in {\mathbb {R}}^{m \times r}$ is the singular value decomposition (SVD) matrix. The eigenvalues and eigenvectors obtained by decomposing *A* into eigenvalues correspond to the parameters of the damped vibration. Let ${\varvec{D}}_{\varvec{\mu }} = \text {diag} \left( \mu _1, \mu _2, \ldots , \mu _r\right)$ be a diagonal matrix of eigenvalues $\mu$ of *A*. We also consider the matrix $\varvec{\Phi } = {\varvec{P}} {\varvec{W}}$, which is a matrix ${\varvec{W}}$ with the eigenvectors of *A* aligned in the column direction and projected by ${\varvec{U}}$. Note that each column is $\left\{ \varvec{\phi } _ 1, \varvec{\phi }_2, \ldots , \varvec{\phi }_r\right\} , \; \varvec{\phi } \in {\mathbb {C}}^M$. Because *A* is a transition matrix of the time evolution, repeatedly applying *t* actions on the initial state vector ${\varvec{v}}_0$ results in ${\varvec{v}}_t$:

18 $$\begin{aligned} {\varvec{v}}_t&= {\varvec{A}}^t {\varvec{v}}_0 \approx \left( \varvec{\Phi } {\varvec{D}}_{\varvec{\mu }}\varvec{\Phi }^{\dagger }\right) ^t{\varvec{v}}_0 = \sum _{i = 1}^{r} \alpha _i \varvec{\phi }_i \mu _i^t. \end{aligned}$$

here, $\varvec{\alpha } = \left( \alpha _1,\alpha _2, \ldots ,\alpha _r\right) ^{\text {T}} \equiv \varvec{\Phi }^{\dagger }{\varvec{v}}_0 \in {\mathbb {C}}^{r}$ and $\varvec{\mu } = \left( \mu _1,\mu _2, \ldots ,\mu _r\right) ^{\text {T}} \in {\mathbb {C}}^{r}$. The DMD is a discrete representation, but it becomes a damped oscillation form if we change it to a continuous representation. First, as a preparation, we convert complex eigenvalues $\mu _ j$ into polar form $\mu _ j = r _ j \exp \left( i \theta _ j\right)$. By this deformation, $\mu _ j$ to the n-th power $\mu _ j ^ n$ is $\mu _ j ^ n = \exp \left( n \ln r _ j\right) \exp \left( i n \theta _ j\right)$. Next, we introduce a continuous time *t*. Assuming that the initial time of the time series data is $t _ 0$ and that the time interval $\Delta t$ is constant, the relationship between the time step *n* and the time *t* is $n = \left( t-t _ 0\right) / \Delta t$. Now, $\mu _ j ^ n$ is expressed using *t* as follows

19 $$\begin{aligned} \mu _j^n = R_j \exp \left( \frac{\ln r_j}{\Delta t} t\right) \exp \left( i \frac{\theta _j}{\Delta t} t\right) \end{aligned}$$

where $R_j$ is $R_j = \mu _j^{-t_0 / \Delta t}$. We rewrite this equation in the form of damped oscillation as follows

20 $$\begin{aligned} \mu _j^n = R_j \exp \left( - {\gamma _j} t\right) \left( \cos \nu _j t + i \sin \nu _j t\right) . \end{aligned}$$

The relationship between ${\tilde{\gamma }}_j$ and ${\tilde{\nu }}_j$ is as follows.

21 $$\begin{aligned} {\tilde{\gamma }}_j = - \frac{\ln r_j}{\Delta t}, \; {\tilde{\nu }}_j = \frac{\theta _j}{\Delta t}. \end{aligned}$$

The ${\tilde{\gamma }}_ j$ corresponds to the damping rate and the ${\tilde{\nu }}_ j$ corresponds to the frequency of vibration. Therefore, the Eq. (18) is rewritten as follows.

22 $$\begin{aligned} {\varvec{v}}_t = \sum _{j = 1}^r {\varvec{R}}_j \exp \left( - {{\tilde{\gamma }}_j} t\right) \left( \cos {\tilde{\nu }}_j t + i \sin {\tilde{\nu }}_j t\right) . \end{aligned}$$

Given that only the real part is observed,

23 $$\begin{aligned} {\varvec{v}}_t = \sum _{n} \exp \left( - {{\tilde{\gamma }}_n} t\right) \left( {\tilde{{{\varvec{a}}}}}_{n} \cos {\tilde{\nu }}_n t + {\tilde{{\varvec{b}}}}_{n} \sin {\tilde{\nu }}_n t \right) . \end{aligned}$$

here, ${\tilde{{\varvec{a}}}}_{\nu } = \; \text {Re} \left( {\varvec{R}}_{ \nu }\right) , \; {\tilde{{\varvec{b}}}}_{ \nu } = - \text {Im} \left( {\varvec{R}}_ {\nu }\right)$. This is consistent with the form of Eq. (10). The damping rate $\gamma$ in Eq. (10) corresponds to the damping rate of DMD modes ${\tilde{\gamma }}$. The frequency $\nu$ in Eq. (10) corresponds to the frequency of DMD modes ${\tilde{\nu }}$. In the same manner, the coefficients of oscillations *a* and *b* in Eq. (10) correspond to the coefficients of the DMD modes $\tilde{a}$ and $\tilde{b}$. Thus, each parameter of the damped vibration can be calculated by DMD.
